# Molecular mechanism of cell ferroptosis and research progress in regulation of ferroptosis by noncoding RNAs in tumor cells

**DOI:** 10.1038/s41420-021-00483-3

**Published:** 2021-05-12

**Authors:** Bumin Xie, Yuan Guo

**Affiliations:** Department of Obstetrics and Gynecology, Key Laboratory for Major Obstetric Diseases of Guangdong Province, The Third Affiliated Hospital of Guangzhou Medical University, Guangzhou, 510150 China

**Keywords:** Gene regulation, Cancer genetics

## Abstract

Ferroptosis is a newly identified form of nonapoptotic regulated cell death characterized by iron-dependent accumulation of lipid reactive oxygen species. Morphologically and biochemically different from known types of cell death and apoptosis, ferroptosis promotes nervous system diseases, renal failure, ischemia–reperfusion injury, and the treatment of tumors. It could be induced by several mechanisms, including inhibition of glutathione peroxidase 4, lack of cysteine, and peroxidation of polyunsaturated fatty acids, but could be inhibited by iron chelators, lipophilic antioxidants, and some specific inhibitors. Ferroptosis is found to be closely related to the tumorigenesis, invasion, and metastasis of tumors. Noncoding RNAs (ncRNAs), including long noncoding RNAs (lncRNAs), microRNAs, and circular RNAs, do not encode proteins. NcRNAs are found to be capable of regulating the molecular mechanism of ferroptosis in tumor cells post transcription. Ferroptosis provides a new method for cancer treatment. Although several studies have confirmed the important role of ferroptosis in cancer treatment, its specific affecting mechanism is unclear. Here we reviewed the molecular mechanism of ferroptosis in tumor cells and the relationship between ferroptosis and the three important ncRNAs.

## Facts

- Ferroptosis is a complex and sophisticated process of cell death that is controlled by multiple factors.
- Noncoding RNA plays an important role in many biological processes of tumorigenesis and development.
- Noncoding RNA plays a very important role in the life activities of tumor cells and plays an irreplaceable role in the regulation of ferroptosis mechanism of tumor cells.

## Open questions

- Ferroptosis, as a newly discovered form of cell death, plays an important role in diseases, such as Parkinson’s disease and tumor. Does it play a role in normal growth and development?
- Are there any new forms of cell death that need to be discovered or named?
- Ferroptosis has a significant effect on the proliferation, death, and metabolism of tumor cells. Could it become a new target for clinical treatment?

## Introduction

Cell death marks the end of cell life under both physiological and pathological conditions. Cell deaths are generally categorized into necrosis and apoptosis. Studies have reported that programmed death modes, including autophagy, oncogenesis, and necrotizing apoptosis, have been found to exhibit unique biological processes and pathophysiological characteristics different from cell necrosis and apoptosis. In 2012, Dixon^1^ first put forward the conception of ferroptosis, which is an iron-dependent, nonapoptotic cell death mode, and the main manifestation is lipid reactive oxygen species (ROS) aggregation. Erastin, a new model of nonapoptotic and non-necrotic cell death, can induce ferroptosis by inhibiting the delivery of cystine in cells, resulting in inactivation of glutathione peroxidase 4 (GPX4) and the depletion of glutathione (GSH). Physiologically, GPX4 transforms lipid peroxides into non-toxic lipid alcohol, thus suppressing toxic side effects of lipid peroxide on cells. However, GPX4 inhibitors (erastin or RSL3) can cause large accumulation of lipid peroxidation products by inhibiting the activity of GPX4 in cells, which leads to cell ferroptosis^2,3^. Ferroptosis is different from necrosis, autophagy, and apoptosis in both morphology and function: it does not exhibit the typical morphological characteristics of necrosis or traditional apoptosis, including cytoplasmic swelling, cell contraction, cell rupturing, apoptotic body formation, and cytoskeleton disintegration; different from autophagy, it does not include forming a classical closed bilayer structure, which is a specific structure named autophagic vacuoles. Morphologically, ferroptosis is presented as the atrophy of mitochondria, with increased membrane density and reduced mitochondrial cristae^4^. Research has demonstrated that ferroptosis plays a regulatory role in disease occurrence and process. Ferroptosis has become a research focus for disease treatment and prognosis.

## The conception of ferroptosis

Ferroptosis is iron-dependent programmed cell death. Iron overload can lead to the abnormal activation of the mitochondrial oxidative phosphorylation pathway and produce high levels of ROS when ATP is produced. When ROS concentration exceeds the scavenging level of the antioxidant system, it can oxidize unsaturated fatty acids on the cell membrane, form lipid peroxides, and directly or indirectly damage the structure and function of cells. This newly discovered cell death process is called ferroptosis^1,5,6^. It is firmly linked to GSH metabolism, iron metabolism, and lipid peroxidation. Therefore, transferrin receptor 1 (TFR1), ferritin, cystine/glutamic acid reverse transporter (system Xc-), GPX4, and lipoxygenase (LOX) are involved in the occurrence of ferroptosis.

## Iron metabolism affects the mechanism of ferroptosis

**Fig. 1:**
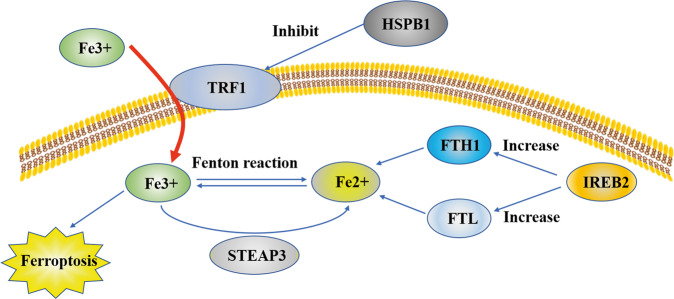
Fe^2+^ is transported into cells through TFR1. Ferritin and its related genes, namely, ferritin heavy chain 1 (FTH1) and ferritin light chain (FTL), regulate the iron ion storage. Heat shock protein B1 (HSPb1) inhibits TFR1 expression to reduce the intracellular iron concentration. Therefore, ferroptosis would be inhibited by overexpression of HSPb1. Iron response element binding protein 2 (IREB2), a major transcription factor involved in iron metabolism, can significantly increase FTL and FTH1 expression, and thus inhibit erastin-induced ferroptosis. The main mechanism of biological toxicity of iron ions is mediated by the classical Fenton reaction between Fe^3+^ and Fe^2+^, which produces hydroxyl radicals that can damage DNA, lipids, and proteins, and result in ferroptosis.

Iron is the most abundant and indispensable trace element in human body and participates in important physiological and biochemical functions. Fe^2+^ is transported into cells through TFR1, then ferritin and its related genes, namely, ferritin heavy chain 1 (FTH1) and ferritin light chain (FTL), regulate the iron ion storage. Heat shock protein B1 (HSPb1) inhibits TFR1 expression, which could reduce the intracellular iron concentration. Therefore, ferroptosis would be inhibited by overexpression of HSPb1^7^. Furthermore, iron response element binding protein 2, a major transcription factor involved in iron metabolism, can significantly increase FTL and FTH1 expression, thus inhibiting erastin-induced ferroptosis^8^. The main mechanism of biological toxicity of iron ions is mediated by the classical Fenton reaction between Fe^3+^ and Fe^2+^, which produces hydroxyl radicals that can damage DNA, lipids, proteins, etc.^9^ (Fig. 1). Dietary iron is mainly absorbed in the duodenum and upper jejunum as Fe^3+^, and after that, Fe^3+^ enters the blood with the help of transferrin. A part of Fe^3+^ in the blood is transported into cells by binding with the transferrin receptor on the cell membrane and is reduced to Fe^2+^ by the metal reductase STEAP3 in the endoplasmic reticulum. Fe^2+^ needs to be released into the cytoplasmic labile iron pool with the help of the solute carrier family 11A2/divalent metal transporter 1 (SLC11A2/DMT1). Excess iron can be transported to the circulation by the iron pump SLC11A2/DMT1 or stored as ferritin.

When dealing with different kinds of ferroptosis activators, intracellular Fe^3+^ levels will increase, and various protein transporters related to iron metabolism, such as ferritin and transferrin receptor, are rearranged under the ferroptosis process^1,10^. Knockout of the transferrin receptor gene or upregulation of cytoplasmic ferritin can inhibit iron overload and ferroptosis^11,12^. However, knockout of SLC11A3, which blocks iron transport, aggravates erastin-induced ferroptosis in neuroma cells^13^. Autophagy can also regulate ferroptosis sensitivity by affecting iron metabolism, and ferritin selective autophagy can increase ferroptosis sensitivity^11,12,14^. Other proteins that affect iron metabolism, such as CISD1, can also affect ferroptosis sensitivity.

## Lipid metabolism and ROS accumulation regulate ferroptosis

**Fig. 2:**
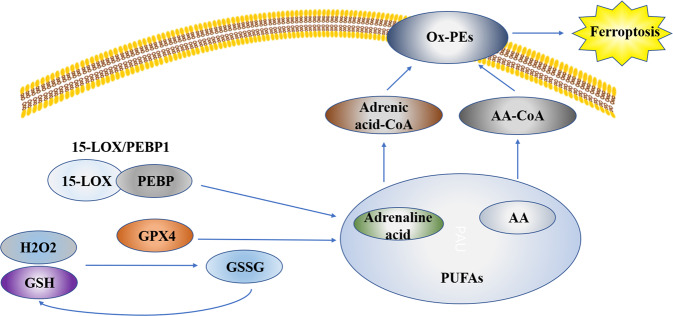
Ferroptosis is induced by excessive oxidation of phospholipids containing polyunsaturated fatty acids (PUFAs) in the cell membrane. Arachidonic acid (AA) and adrenic acid in PUFAs are the key components that induce ferroptosis. These PUFA coenzyme-a derivatives, or oxidized phosphatidylethanolamines (ox-PEs), are esterified by ACSL4. AA and adrenic acid are converted into arachidonic acid-CoA (AA-CoA) and adrenic acid-CoA. Free PUFAs are the preferred substrates for LOXs. LOXs combine with PEBP1 to form the 15-LOX/PEBP1 complex and initiates ferroptosis. Glutathione peroxidases (GPXs) use GSH as a cofactor to catalyze the decomposition of hydrogen peroxide; 15-LOX/PEBP1 and GPX4 regulate the oxidation and antioxidation of esterified fatty acids. When the activity or expression of GPX4 decreases, ROS content increases, long-chain PUFAs on the membrane are oxidized, and ferroptosis is induced.

Cell ferroptosis is caused by the imbalance of lipid oxidation metabolism. In normal cells, lipid oxidation and reduction are in a dynamic equilibrium. Because of exogenous factors or carcinogenesis, intracellular homeostasis is disturbed, and the expression level of lipid oxidation related genes is upregulated or that of lipid reduction-related genes is inhibited, which leads to considerable accumulation of lipid oxide in cells and ferroptosis^15^. Studies have shown that ferroptosis is induced by excessive oxidation of phospholipids containing polyunsaturated fatty acids (PUFAs) in the cell membrane^15^. Lipid peroxidation stress and cell membrane damage play a key role in this induction process. In particular, PUFAs are more likely to form lipid peroxide and induce ferroptosis^16^. A lipomics study found that arachidonic acid (AA) and adrenic acid in PUFAs are the key contributors to induction of ferroptosis^17^. These PUFA coenzyme-a derivatives, or oxidized phosphatidylethanolamines, are esterified by ACSL4. AA and adrenic acid are converted into arachidonic acid-CoA and adrenic acid-CoA, respectively, and participate in the synthesis of phosphatidylethanolamine or phosphatidylinositol and other negatively charged membrane phospholipids, which are incorporated into the cell membrane. LOX is a class of non-heme iron enzymes, which catalyzes the peroxidation of unsaturated fatty acids and affects ferroptosis^18^. Free PUFAs are the preferred substrates for LOXs. The knockout of LOX can reduce erastin-induced ferroptosis injury^19^. Phosphotidylethanolamine-binding protein 1 (PEBP1) is a small scaffold protein, also known as RAF1 kinase inhibitor protein. It can bind with RAF1 and inhibits the RAF1-mediated kinase cascade under steady states. LOXs combine with PEBP1 to form the 15-LOX/PEBP1 complex; simultaneously, allosteric regulation forms the ferroptosis marker signal 15-HpETE-PE, thus initiating ferroptosis^20^. ROS is a molecule that contains partly reduced oxygen. Examples include superoxide (O_2_−), peroxides (H_2_O_2_ and ROOH), and free radicals (HO· and RO·)^19^. In cells, GPXs use GSH as a cofactor to catalyze the decomposition of hydrogen peroxide and peroxide into H_2_O and corresponding alcohols. Under normal conditions, 15-LOX/PEBP1 and GPX4 regulate the oxidation and antioxidation of esterified fatty acids. However, when the two processes are not balanced, long-chain PUFAs on the membrane are oxidized, especially through ferroptosis induced by RSL3 or other factors. If the activity or expression of GPX4 decreases, ROS content increases and ferroptosis is induced (Fig. 2).

**Fig. 3:**
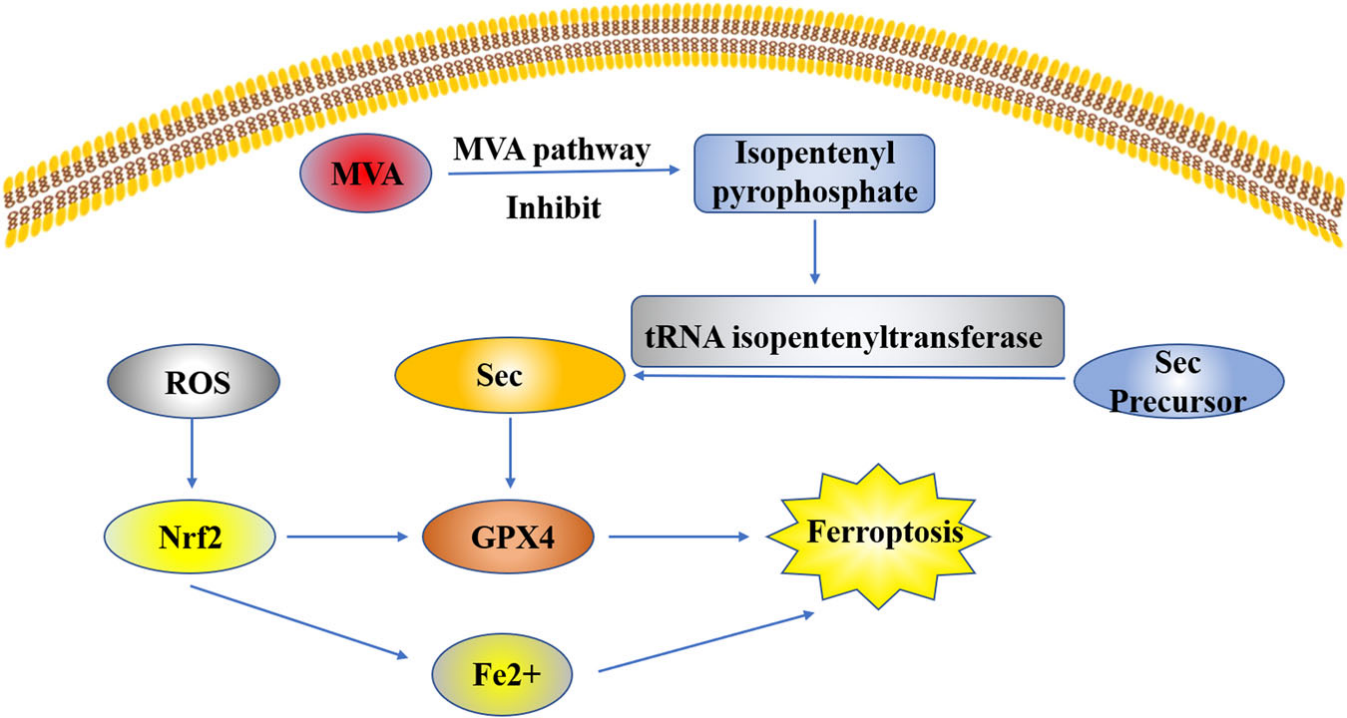
The Nrf2-Keap1 system can resist exogenous and endogenous oxidative damage and regulate the expression of human antioxidant protein. Nrf2 can regulate Fe^2+^ in cells. When stimulated by ROS or induced by electrophiles, Nrf2 changes its molecular conformation and activates downstream antioxidant enzymes to inhibit oxidation and cell ferroptosis. The expression of GPX4 gene is mediated by Nrf2 transcription, and GPX4 transforms highly toxic lipid hydrogen peroxide into non-toxic aliphatic alcohols and decomposes hydrogen peroxide into water. It is a selenoprotein that can effectively repair oxidative damage to unsaturated fatty acids in mammals. The biosynthesis of GPX4 is controlled by Selenocysteine tRNA (Sec) in the human body. Sec undergoes a lipid modification (isopentenylation). The modified enzyme, namely, tRNA isopentenyltransferase, uses isopentenyl pyrophosphate (a product of the mevalonate pathway) as donor. The mevalonate pathway inhibitors interfere tRNA maturation and GPX4 biosynthesis to regulate ferroptosis.

The Nrf2-Keap1 system, NF-E2-related factor 2 (Nrf2)-kelch-like ECH-associated protein 1 (Keap1), can not only regulate cellular homeostasis but also resist exogenous and endogenous oxidative damage and regulate the expression of human antioxidant proteins, which is the key redox pathway. Nrf2 can regulate Fe^2+^ in cells. It remains inactive under normal conditions, but when stimulated by ROS or induced by electrophiles, it changes its molecular conformation and activates downstream antioxidant enzymes to inhibit oxidation and cell ferroptosis^21^. The expression of GPX4 gene is mediated by Nrf2 transcription, and GPX4 transforms highly toxic lipid hydrogen peroxide into non-toxic aliphatic alcohols and decomposes hydrogen peroxide into water. It is a selenoprotein that can effectively repair oxidative damage to unsaturated fatty acids in mammals^17^. The biosynthesis of all 25 selenoproteins is controlled by Selenocysteine tRNA (Sec) in the human body^22^. Sec undergoes a lipid modification (isopentenylation), which is necessary for its effective incorporation into selenoproteins, at specific adenine sites during maturation. The modified enzyme, namely, tRNA isopentenyltransferase, uses isopentenyl pyrophosphate (a product of the mevalonate pathway) as a donor. The mevalonate pathway inhibitors were reported to interfere with tRNA maturation and GPX4 biosynthesis to regulate ferroptosis^23^ (Fig. 3).

## System Xc- and GSH

**Fig. 4:**
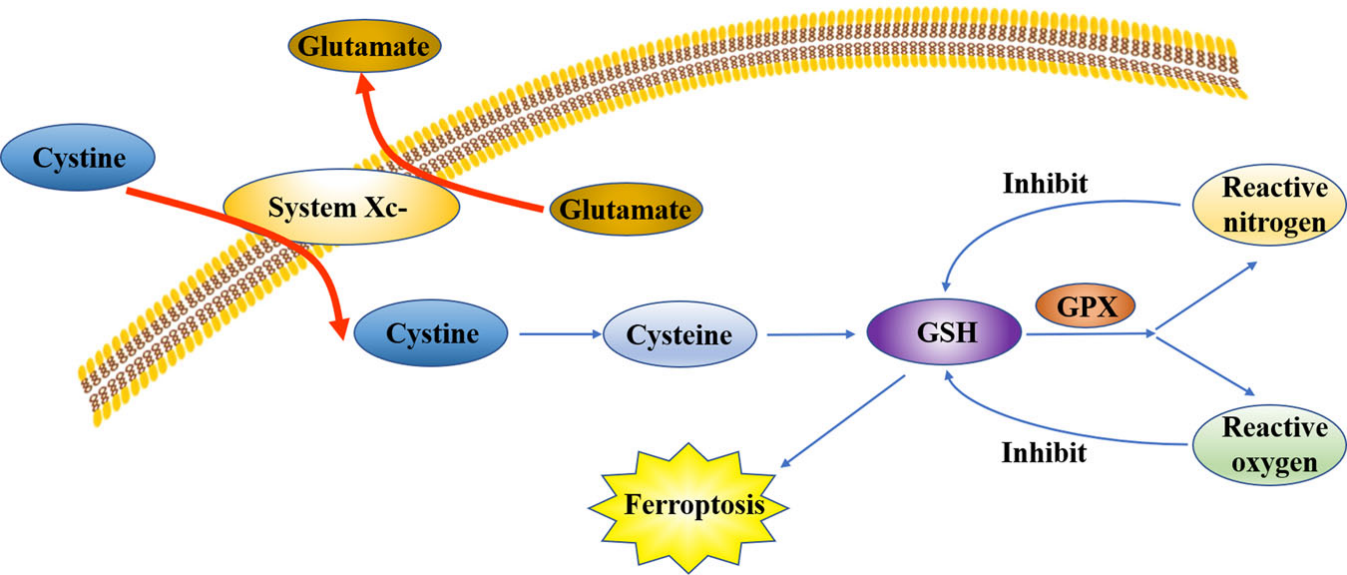
Ferroptosis could be induced by the inhibition of system Xc-, which is an important antioxidant system and comprises two subunits, namely, solute carrier family 3 member 2 (SLC3A2) and solute carrier family 7 member 11 (SLC7A11). It transports glutamate out of the cell and transports cystine into the cell simultaneously. Cystine reduced to cysteine by system Xc- participates in GSH synthesis. Gamma-glutamyl-l-cysteine-l-glycine (GSH) is an antioxidant and scavenges free radicals. In a reaction catalyzed by glutathione peroxidase (GPX), GSH reduces reactive oxygen and reactive nitrogen, thus inhibiting GSH synthesis, which result in ferroptosis.

**Fig. 5:**
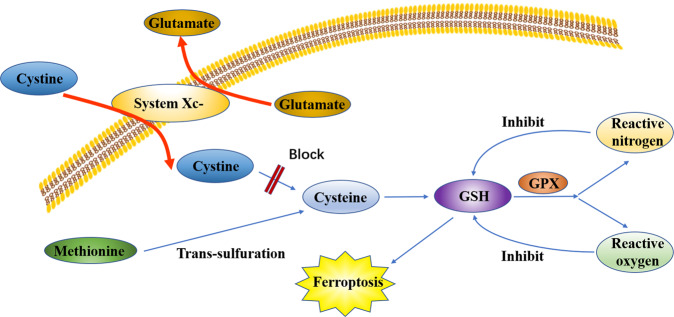
Methionine can synthesize cysteine through *trans*-sulfuration. When intracellular system Xc- was inhibited, the cells continued to synthesize cysteine. Therefore, the ferroptosis inducer, which negatively regulates system Xc-, cannot kill cells effectively and completely.

Inhibition of system Xc- was found to be a cause of cell ferroptosis^1^. System Xc-, an important antioxidant system, is a transporter located in the cell membrane and comprises two subunits, namely, solute carrier family 3 member 2 and solute carrier family 7 member 11 (SLC7A11). It transports glutamate out of the cell and cystine into the cell simultaneously^24^. Cystine reduced to cysteine by system Xc- participates in GSH synthesis^25^. Gamma-glutamyl-l-cysteine-l-glycine (GSH) is a tripeptide comprising glutamic acid, cysteine, and glycine. GSH is an antioxidant and scavenges free radicals. In a reaction catalyzed by GPX, GSH reduces reactive oxygen and reactive nitrogen, thus inhibiting GSH synthesis, which results in oxidative damage and cell death. This classic cellular oxidative stress pathway has been shown to be associated with ferroptosis (Fig. 4). Methionine can also synthesize cysteine through *trans*-sulfuration. When the intracellular system Xc- is inhibited, the cells continue to synthesize cysteine. Therefore, the ferroptosis inducer, which negatively regulates system Xc-, cannot kill cells effectively. Hayano et al.^26^ found that reducing the expression of cysteine tRNA synthetase by interfering RNA can activate the *trans*-sulfuration pathway, thus reducing the sensitivity of cells to ferroptosis inducers. Glutamate and glutamine play a significant role in the regulation of ferroptosis (Fig. 5)^27^. System Xc-, a main system on the cell membrane for maintaining glutamate and cystine homeostasis, pumps out one molecule of glutamic acid and simultaneously pumps in a molecule of cystine. Extracellular glutamate levels simultaneously regulate the activity of system Xc-^28^. Studies have shown that high concentrations of glutamate can lead to the inactivation of system Xc-. Findings from a study revealed the mechanism of cytotoxicity of high concentration glutamate in cells of the central nervous system^29^. Therefore, extracellular glutamate accumulation may induce ferroptosis under physiological conditions.

## Other mechanisms for ferroptosis induction

**Fig. 6:**
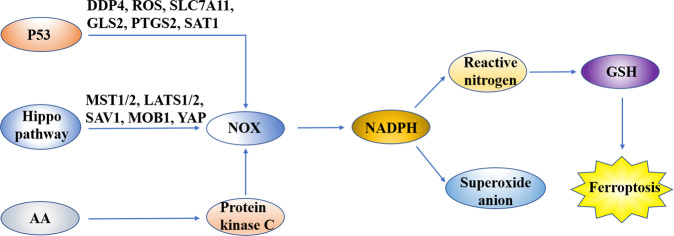
Nicotinamide adenine dinucleotide phosphate (NADPH) can maintain the reduced state of GSH, which could regulate ferroptosis. NADPH oxidase (NOX) is an enzyme complex, which consumes NADPH to produce superoxide anion and oxidative free radical. Overexpression of NOX can lead to the depletion of NADPH and upregulate the level of oxidative free radicals, which significantly increases the sensitivity to ferroptosis. NOX function can be regulated in three ways. First, p53 can inhibit ferroptosis of CRC cells by binding dipeptidyl peptidase-4 (DPP4), which regulates ferroptosis and lipid metabolism. AA can significantly enhance the level of protein kinase C-mediated phosphorylation of NOX. However, the increase in NOX phosphorylation increases the amount of oxidative free radicals and risk of ferroptosis. The Hippo pathway is mainly composed of the serine/threonine kinase mammalian Ste20-like kinases 1/2 (MST1/2), Salvador family WW domain containing protein 1 (SAV1), large tumor suppressor 1/2 (LATS1/2), MOB kinase activator 1 (MOB1), and transcriptional co-activator YAP.

Nicotinamide adenine dinucleotide phosphate (NADPH), a reductase of GSH, can maintain the reduced state of GSH to regulate ferroptosis. Thus, NADPH can be used as a biomarker of cell ferroptosis-inducer sensitivity^30^. Studies have shown that NADPH oxidase (NOX)-mediated oxidation is an important source of oxidative free radicals^1^. NOX is an enzyme complex, which consumes NADPH to produce superoxide anion and oxidative free radicals, which are important for the immune system and cell signal transduction. Overexpression of NOX can lead to the depletion of NADPH and elevate the level of oxidative free radicals, which significantly increases the sensitivity to ferroptosis. By contrast, NOX inhibitors can downregulate NOX expression, thus inhibiting the function of the ferroptosis-inducer erastin. Moreover, ROS produced during ferroptosis were related to an increase in NOX levels^1^. Gao et al.^27^ found that glutamine metabolism was involved in the activation of ferroptosis through the production of ROS. Current research results show that NOX function can be regulated in three ways. First, p53 can inhibit ferroptosis of colorectal cancer (CRC) cells by binding dipeptidyl peptidase-4 (DPP4)^31^, which regulates ferroptosis and lipid metabolism. It has peptidase activity and can degrade various bioactive peptides, but its enzyme activity is not important for ferroptosis. It can bind with NOX1 and mediate ROS production in the DPP4-dependent cell membrane and plasma, resulting in lipid peroxide accumulation and ferroptosis. In 2015, Jiang et al.^32^ first linked p53 with ferroptosis, believing that p53 can inhibit SLC7A11 in a transcription-dependent manner to induce ferroptosis in cells. In addition to SLC7A11, some p53 target genes promote ferroptosis, including glutamine 2 (GLS2), prostaglandin endoperoxide synthase 2, and spermidine/spermine N1 acetyltransferase 1. The regulation of p53 is complex and delicate in cells: different cell types, different types of stress factors, and even the same stress factor at different intensities may stimulate different p53 signaling pathways and lead to different cell fates^33^. Studies have shown that AA can significantly enhance the level of protein kinase C-mediated phosphorylation of NOX^34^. However, the increase in NOX phosphorylation increases the amount of oxidative free radicals and risk of ferroptosis. The Hippo pathway is mainly composed of the serine/threonine kinase mammalian Ste20-like kinases 1/2, Salvador family WW domain containing protein 1, large tumor suppressor 1/2, MOB kinase activator 1, and transcriptional co-activator YAP^35–38^. Luo et al.^39^ found that miR-137 can modulate ferroptosis by combining the 3’ untranslated region (UTR) region of glutamine transporter SLC1A5 mRNA negatively (Fig. 6).

## Noncoding RNAs (ncRNAs) regulate ferroptosis in tumor cells

RNA, important biological macromolecules widely present in cells, can be divided by their functions into two categories, namely, coding RNAs and ncRNAs. RNAs are responsible for information transmission and gene regulation and are related to the occurrence of many human diseases. Herein, we reviewed ncRNAs that include microRNA (miRNA), long-chain ncRNA (lncRNA), and circular RNA (circRNA), which play regulatory roles in ferroptosis.

## LncRNA regulates ferroptosis

**Fig. 7:**
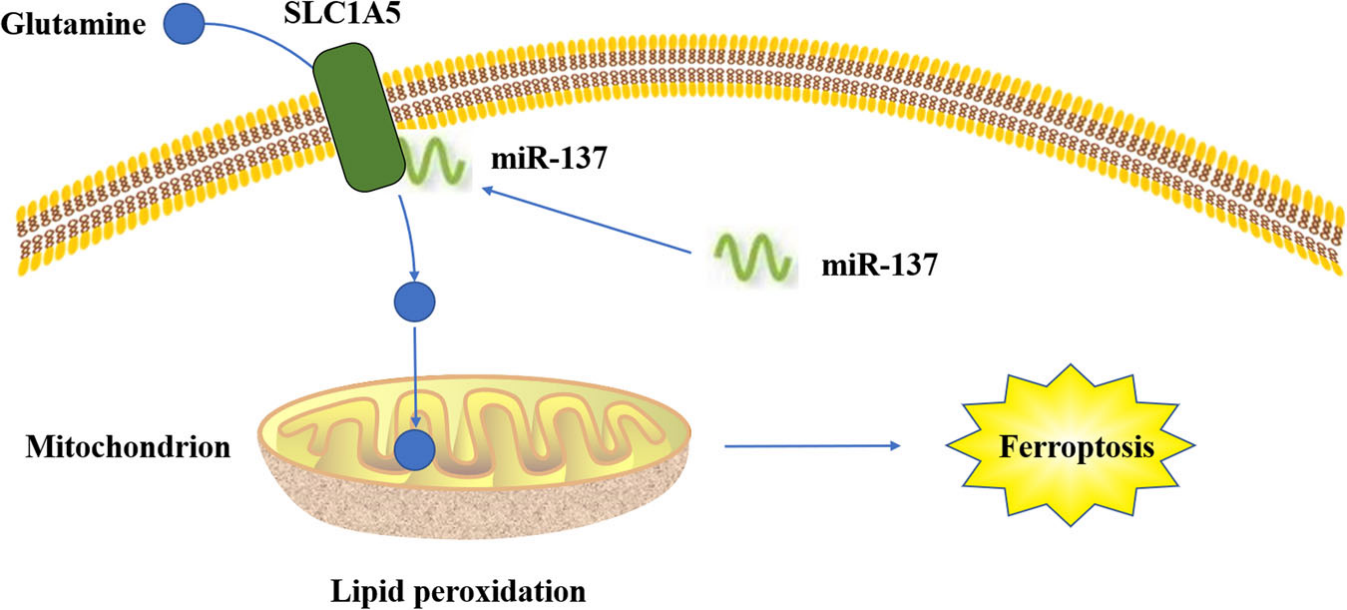
miR-137 suppresses erastin-induced ferroptosis by directly targeting SLC1A5. MiR-137 prevents glutamine from entering cells by directly binding to SLC1A5. Glutamine is an important antioxidant component in cells. Due to the lack of glutamine, lipid peroxidation occurs in mitochondria, which eventually leads to iron death.

LncRNA is a ncRNA with a length of >200 nucleotides. LncRNA is mainly transcribed by RNA polymerase II, which does not have an obvious open reading frame. LncRNA is involved in many bioprocesses, such as cell growth, anti-apoptosis, migration, and invasion. The functions of lncRNA, including histone modification, chromatin remodeling, transcriptional activation, transcriptional interference, nuclear transport, and cell cycle regulation, depend on their subcellular localization^40–44^. LncRNA is also involved in regulation of ferroptosis. It was found that, in tumor, cytoplasmic lncRNA P53RRA is downregulated and interacts with Ras-GTPase activating protein binding protein 1 (G3BP1) to transfer p53 from the G3BP1 complex, resulting in p53 retention in the nucleus, leading to cell cycle arrest, ferroptosis, and apoptosis^45^. GABBB1 and its antisense chain lncRNAGAPB1-AS1 can interact in erastin-induced ferroptosis; GAPBB1 and GAPBB1-AS1 can be used as therapeutic targets for liver cancer^46^. Lymphoid-specific helicase (LSH) can increase the transcription of SLC7A11 after the recruitment to the promoter regions of SLC7A11, and LINC00618 can reduce the expression of LSH, thus inhibiting ferroptosis^47^ (Fig. 7).

## MiRNA regulates ferroptosis

**Fig. 8:**
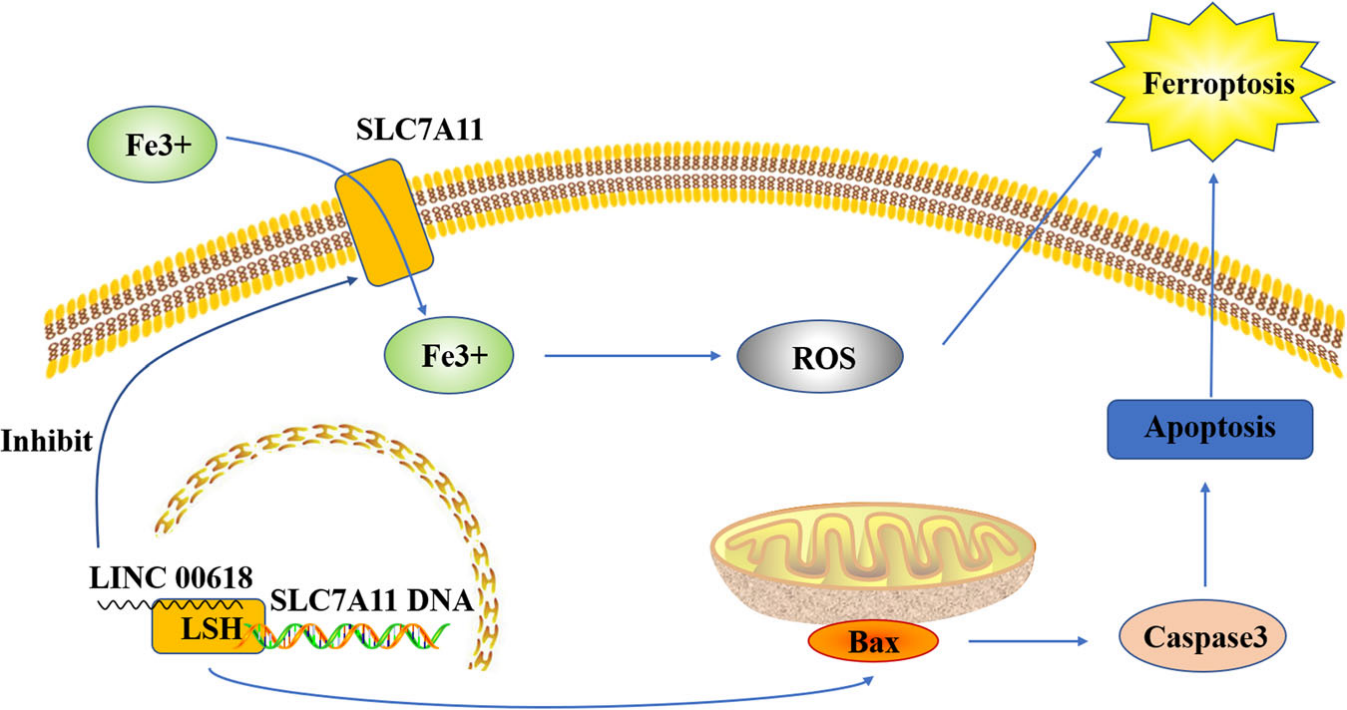
LINC00618 promotes apoptosis by increasing the levels of BCL2-associated X (BAX) and cleavage of caspase-3. LINC00618 also accelerates ferroptosis by increasing the levels of lipid reactive oxygen species (ROS) and iron, two surrogate markers of ferroptosis, and decreasing the expression of solute carrier family 7 member 11 (SLC7A11).

MiRNA is a type of noncoding single-stranded RNA molecules with a length of 20–24 nucleotides in cells, which are encoded by endogenous genes and play important regulatory roles. MiRNAs, first found in *Caenorhabditis elegans* by Lee in 1993^48^, can regulate multiple metabolic pathways in humans at the level of translation and transcription and play a key role in regulating tumor cell growth, migration, invasion, and inducing resistance to chemotherapy^49^. They regulate gene expression by binding to the 3’ UTR of target mRNA transcripts at the post-transcriptional level negatively^50^. The binding of miRNAs to target mRNAs results in translation inhibition or decrease of mRNA stability. MiRNAs regulate different biological processes, including cell proliferation, cell differentiation, apoptosis, drug resistance, and fat metabolism^51^. They can also regulate ferroptosis in tumor cells: MiR-137 regulates ferroptosis by targeting the glutamine transporter SLC1A5 in melanoma^39^ (Fig. 8); MiR-9 regulates ferroptosis in melanoma by targeting glutamic oxaloacetate transaminase^52^; MiR-214 plays an active role in enhancing erastin-induced ferroptosis by destroying the ATF4 imbalance in hepatoma cells^53^. Similarly, in glioma cells, ATF4 gene knockout increases the sensitivity of tumor cells to RSL3- and erastin-induced ferroptosis to inhibit tumor growth^54^. This study suggests that the miR-214–ATF4 axis may be a potential therapeutic target for liver cancer with ferroptosis. It was reported that physcion 8-*O*-β-glucopyranoside promotes ferroptosis and antitumor activity in vivo and in vitro by regulating the miR-103a-3p/GLS2 axis, indicating a new approach to treat gastric cancer^55^. The negative regulatory effect of TP53 on ferroptosis in human CRC cells has been studied extensively. MiR-150-5p limits ferroptosis by regulating the tumor-suppressor TP53, which blocks the activity of DPP4. TP53 and SLC7A11 can induce ferroptosis and are important for the prognosis and treatment of CRC^31,56^. Mir-7-5p also plays a role in ferroptosis by downregulating mitoferritin and reduces Fe^2+ ^^57^.

## CircRNA regulates ferroptosis

CircRNAs are a special class of ncRNAs without the protein-coding function, formed by the 3′ and 5′ ends of mRNA, and are mainly produced by the intron or exon through reverse splicing or a lasso intron^58^. Different from typical linear RNAs, circRNAs are not affected by RNA exonucleases due to the closed loop structure and are more stable and difficult to degrade; they can bind miRNAs competitively and act as a miRNA sponge to play a role^59^. CircRNAs can affect the stability and transcription of miRNAs at the post-transcriptional level by changing the expression level of miRNA-related target genes^60,61^. It was found that circRNA is involved in mitogen-activated protein kinase/extracellular regulation of protein kinase, phosphatidylinositol 3 kinase/protein kinase B, and Wnt/β-catenin cell signal transduction pathways and participates in the regulation of ferroptosis in tumor cells^62,63^. CircRNA cIARS regulate ferroptosis in hepatocellular carcinoma cells by interacting with the RNA-binding protein ALKBH5^64^.

## Interaction between ncRNAs that regulate ferroptosis in tumor cells

**Fig. 9:**
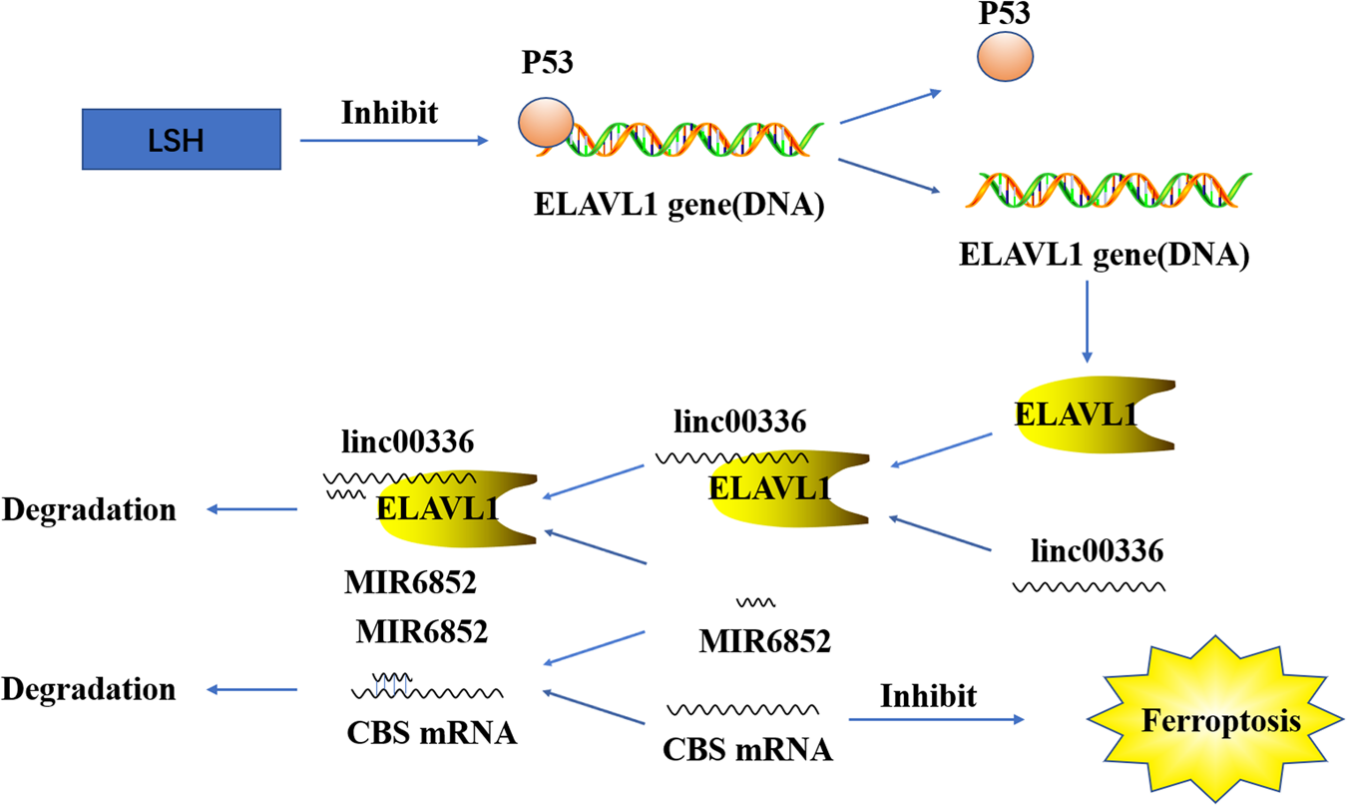
Long noncoding RNA LINC00336 inhibits ferroptosis by functioning as a competing endogenous RNA. LSH induces ELAVL1 expression through the inactivation of p53 and ELAVL1 enhances LINC00336 levels through transcriptional regulation by interacting with LINC00336. Then LINC00336 combines with MIR6852 as a ceRNA, which evaluates the mRNA level of cystathionine-β-synthase (CBS), stimulating cell proliferation, colony formation, and tumor formation, and inhibiting ferroptosis in lung cancer.

**Fig. 10:**
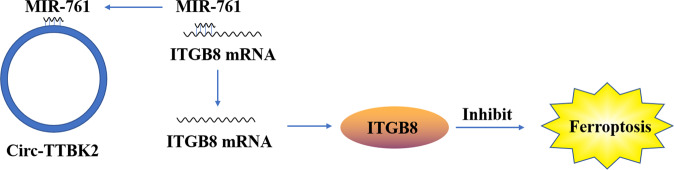
Circ-TTBK2 inhibits ferroptosis by competing with ITGB8 mRNA. ITGB8 was a target of miR-761. Circ-TTBK2 and ITGB8 were expressed at high levels, whereas miR-761 was downregulated in glioma tissues and cells. ITGB8 could regulate tumor growth and metastasis. miR-761, targeted by circ-TTBK2 and ITGB8 mRNA, regulated the aggressive behaviors and ferroptosis.

MiRNAs can regulate gene expression by competitively binding lncRNA and circRNA. Circl4r acts as a promoter and ferroptosis inhibitor in liver cancer through miR-541-3p/GPX4^65^. The metallothionein 1D pseudogene/miR-365a-3p/NRF2 axis could be used as a novel strategy to increase erastin-induced ferroptosis in non-small cell lung cancer cells (NSCLCs)^66^. By regulating the miR-326/C-C chemokine motif ligand 5 axis, circABCB10 is silenced to promote ferroptosis and apoptosis in rectal cancer cells, providing a potential therapeutic target for treating rectal cancer^67^. Previous research has shown that MIR6852 inhibits tumor cell growth by promoting ferroptosis. Cystathionine-β-synthase (CBS) is an alternative marker of ferroptosis. Linc00336, as an endogenous sponge of miRNA 6852, regulates the expression of CBS and then regulates ferroptosis in tumor cells^68^ (Fig. 9). LncRNA-PVT1 regulates ferroptosis through miR-214-mediated TFR1 and TP53 expression. Moreover, positive feedback loops of lncRNA-PVT1/miR-214/p53 have been reported^69^. XAV939-induced downregulation of lncRNA MIR503HG may be due to the downregulation of SOX4 by sponge miR1273c, thus inhibiting the progress of NSCLC. Moreover, XAV939 may participate in ferroptosis and inhibit the development of NSCLC through the downregulation of SLC7A11^70^. Circ-TTBK2 regulates the proliferation, invasion, and ferroptosis of glioma cells through activating miR-761 and targeting ITGB8, which may be used as a biomarker for the clinical treatment of glioma^71^ (Fig. 10). In addition, many RNA-binding proteins, such as ZFP36/TTP, ELAVL1/HuR, and RBM10, can regulate ferroptosis in tumor cells^72–74^.

## Conclusion

Cell death is necessary for normal metabolism of the human body. Ferroptosis, a new type of programmed cell death mode caused by excess accumulation of iron-dependent lipid peroxide and ROS, plays an important role in the incidence, development, and treatment of neurological diseases, ischemia–reperfusion injuries, kidney injuries, tumor development, and other diseases. Some progress has been made in understanding the molecular mechanism of ferroptosis, which involves the expression of various molecules and signal pathway components, among which iron, lipid, and amino acid metabolism are key regulatory mechanisms. Additionally, ncRNAs, widely present in cells, is of great significance in tumor occurrence and development, clinical diagnosis, and treatment, as well as prognosis evaluation. Moreover, ncRNA plays a regulatory role in ferroptosis of tumor cells and provides a new direction for the diagnosis and treatment of tumors; however, the specific regulatory mechanism of ncRNAs in the regulation of ferroptosis is yet to be clarified. Further research is needed to provide more insights for the development of comprehensive tumor treatments.

## Supplementary information

Author Contribution Form
