# Supplementary material for: Molecular mechanism of cell ferroptosis and research progress in regulation of ferroptosis by noncoding RNAs in tumor cells
Source: Cell Death Discov. 2021 May 12;7:101. doi: 10.1038/s41420-021-00483-3 (PMC8115351; doi:10.1038/s41420-021-00483-3)
Supplement: Supplementary file 1 — Author Contribution Form [file 41420_2021_483_MOESM1_ESM.pdf]

DECLARATION OF CONTRIBUTIONS TO ARTICLE

ADMC

Manuscript Number:	Journal Name:	
CDDISCOVERY-21-1403	*Cell Death Discovery*	(the 'Journal')

Proposed Title of the Contribution:

Molecular Mechanism Analysis of Cell Ferroptosis and Research Progress of Noncoding RNA Regulating Ferroptosis in Tumor Cells (the 'Contribution)

Author(s):

Yuan Guo,Bumin Xie (the 'Authors')

For all *CDDis*covery articles, each person named as an author in the published version must be able to show he or she has contributed substantially to the article.

Authorship credit should be based on 1) substantial contributions to conception and design, acquisition of data, or analysis and interpretation of data; 2) drafting the article or revising it critically for important intellectual content; and 3) final approval of the version to be published. Authors should meet conditions 1, 2 and 3.

Any person who cannot be shown to have made a substantial contribution to the article cannot be listed as an author in the final version. The name of any person who is deemed to have made a minor contribution can, however, appear in the Acknowledgments section of the article.

Please complete the table below to indicate the contributions of all named authors to the manuscript.

Author Full Name:	Specification of Contribution to the Manuscript:
Yuan Guo	substantial contributions to conception and design, assembled the figure and final approval of the version to be published
Bumin Xie	drafting the article or revising it critically for important intellectual content, provided technical and material support

Please complete the table below to indicate the contributions of all named authors to the figures.

Figure 1:

Yuan Guo assembled the figure

Figure 2:

Yuan Guo assembled the figure

Figure 3:

Yuan Guo assembled the figure

Figure 4:

Yuan Guo assembled the figure

Figure 5:

Yuan Guo assembled the figure

Figure 6:

Yuan Guo assembled the figure

Signed for and on behalf of the Author(s):	Print Name:	Date:
郭元	Yuan Guo	2021-2-14
